# Crucial roles of thymidine kinase 1 and deoxyUTPase in incorporating the antineoplastic nucleosides trifluridine and 2′-deoxy-5-fluorouridine into DNA

**DOI:** 10.3892/ijo.2015.2974

**Published:** 2015-04-20

**Authors:** KAZUKI SAKAMOTO, TATSUSHI YOKOGAWA, HIROYUKI UENO, KEI OGUCHI, HIROMI KAZUNO, KEIJI ISHIDA, NOZOMU TANAKA, AKIKO OSADA, YUKARI YAMADA, HIROYUKI OKABE, KENICHI MATSUO

**Affiliations:** Drug Discovery and Development I, Discovery and Preclinical Research Division, Taiho Pharmaceutical Co., Ltd., Tsukuba, Ibaraki 300-2611, Japan

**Keywords:** TAS-102, trifluridine, tipiracil hydrochloride, 5-fluorouracil

## Abstract

Trifluridine (FTD) and 2′-deoxy-5-fluorouridine (FdUrd), a derivative of 5-fluorouracil (5-FU), are antitumor agents that inhibit thymidylate synthase activity and their nucleotides are incorporated into DNA. However, it is evident that several differences occur in the underlying antitumor mechanisms associated with these nucleoside analogues. Recently, TAS-102 (composed of FTD and tipiracil hydrochloride, TPI) was shown to prolong the survival of patients with colorectal cancer who received a median of 2 prior therapies, including 5-FU. TAS-102 was recently approved for clinical use in Japan. These data suggest that the antitumor activities of TAS-102 and 5-FU proceed via different mechanisms. Thus, we analyzed their properties in terms of thymidine salvage pathway utilization, involving membrane transporters, a nucleoside kinase, a nucleotide-dephosphorylating enzyme, and DNA polymerase α. FTD incorporated into DNA with higher efficiency than FdUrd did. Both FTD and FdUrd were transported into cells by ENT1 and ENT2 and were phosphorylated by thymidine kinase 1, which showed a higher catalytic activity for FTD than for FdUrd. deoxyUTPase (DUT) did not recognize dTTP and FTD-triphosphate (F_3_dTTP), whereas deoxyuridine-triphosphate (dUTP) and FdUrd-triphosphate (FdUTP) were efficiently degraded by DUT. DNA polymerase α incorporated both F_3_dTTP and FdUTP into DNA at sites aligned with adenine on the opposite strand. FTD-treated cells showed differing nuclear morphologies compared to FdUrd-treated cells. These findings indicate that FTD and FdUrd are incorporated into DNA with different efficiencies due to differences in the substrate specificities of TK1 and DUT, causing abundant FTD incorporation into DNA.

## Introduction

**Figure 1:**
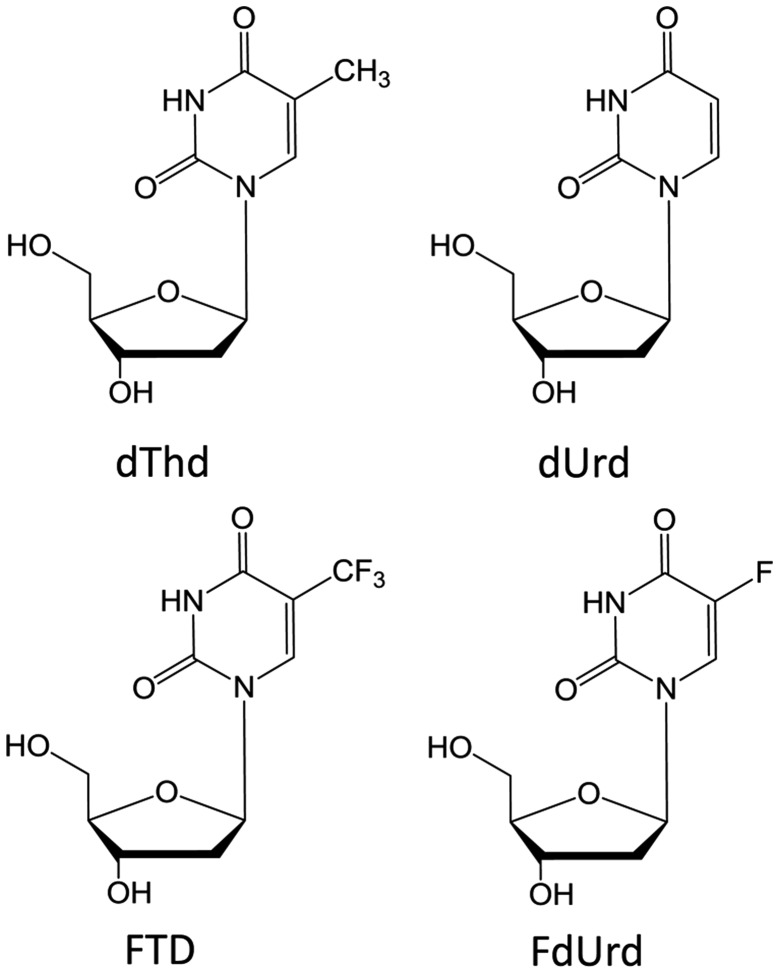
Structures of natural and analog pyrimidine nucleosides.

Because DNA replication strongly depends on the pool of available deoxyribonucleoside triphosphates, the intracellular metabolism of tumor cells adapts to facilitate rapid proliferation. Accordingly, nucleic acid metabolism is one of the most upregulated pathways in tumor tissues (1,2). Thus, the pyrimidine synthesis pathway involving deoxythymidine (dThd; Fig. 1) biosynthesis was recognized in early studies as a target for solid tumor chemotherapy with drugs, such as 5-fluorouracil (5-FU) and/or antifolate agents (3,4).

Thymidylate synthase (TS), the target of 5-FU and antifolate drugs, is highly expressed in various tumor tissues and is the sole *de novo* enzyme of dThd synthesis. TS catalyzes the methylation of deoxyuridine monophosphate (dUMP) to dTMP (5–7). However, the dThd salvage pathway involves multiple factors, such as nucleoside transporters and dThd kinases (TK). TK1 is expressed in the cytoplasm during S phase (8), while TK2 expression is localized to mitochondria and is cell cycle independent (9). TK1 and TS are highly upregulated in various tumor tissues (7) and may serve as potential targets for cancer therapy. However, antitumor agents targeting the dThd salvage pathway have yet to be developed clinically.

Trifluridine (FTD; Fig. 1) is a thymidine-derived nucleoside first synthesized by Heidelberger *et al* in 1964 as an antitumor agent (10), and clinical trials using FTD for monotherapy have been conducted in US (11). However, these trials showed an unexpected toxicity, and FTD was later repurposed as the ocular antiviral drug Viroptic^®^ (12). FTD is well absorbed, but it is easily degraded by the hepatic enzyme thymidine phosphorylase (TP) following oral administration. TAS-102 is an oral combination of FTD and tipiracil hydrochloride (TPI) that prevents FTD degradation by TP (13). Co-administration of TPI and FTD increases the overall FTD concentration in the body, leading to augmented antitumor activity (14).

Recently, TAS-102 treatment showed prolonged survival in patients with metastatic colorectal cancer (mCRC) that were refractory or intolerant to standard chemotherapies including 5-FU, oxaliplatin and CPT-11, in a *KRAS* mutation-independent manner (15). Based on this phase II result, TAS-102 was launched in Japan in May 2014 as an agent for treating unresectable advanced and recurrent colorectal cancers. The antitumor activity of FTD occurs via two distinct mechanisms, namely, TS inhibition by the mononucleotide form of FTD (F_3_dTMP) and DNA incorporation itself (16,17). Previous studies have shown that the mechanism of TS inhibition of FTD is different from that of 5-FU (18,19). Moreover, in the phase II study mentioned above, TAS-102, showed efficacy in patients who were progressive after treatment with 5-FU, confirming that FTD and 5-FU have different mechanisms of cytotoxicity.

TS inhibition by the metabolites of FTD or FdUrd (Fig. 1), a clinically active 5-FU analog, has been described by Reyes and Heidelberger (20). Both nucleosides were reported to be metabolized by dThd salvage pathway, involving the nucleoside transporter family members hENT and TK1 (21–23). However, the DNA incorporation profiles regarding substrate specificities in DNA extension reactions by DNA polymerase were not compared. Moreover, in terms of nucleoside triphosphate specificity during DNA synthesis, deoxyUTPase (DUT) plays an important role in DNA replication and 5-FU sensitivity. DUT functions as a gatekeeper protein to prevent the misincorporation of deoxyuridine-triphosphate (dUTP) into DNA by converting dUTP to dUMP. DUT also converts FdUTP (FdUrd-triphosphate) to FdUMP (FdUrd-monophosphate) and prevents FdUTP misincorporation, such that high DUT expression causes 5-FU resistance (24).

These phenomena indicate that the incorporation of 5-FU metabolites and dUTP into DNA are important for 5-FU cytotoxicity, but investigations regarding the DNA incorporation profile of FTD have been limited (25). Therefore, we studied the levels of FTD and FdUrd incorporation into DNA, as well as the substrate specificities of hENT family members (hENT1 and hENT2), TK1, DUT and DNA polymerase α.

## Materials and methods

### Chemical and reagents

FTD was obtained from Yuki Gosei Kogyo Co., Ltd. (Tokyo, Japan). TPI was synthesized at Junsei Chemical Co., Ltd. (Tokyo, Japan). dThd, FdUrd and dUrd were purchased from Wako Pure Chemical Industries, Ltd. (Osaka, Japan). [5-methyl-^3^H] dThd (25.0 Ci/mmol), [6-^3^H] dUrd (19 Ci/mmol), [6-^3^H] FdUrd (13.5 C i/mmol), and [6-^3^H] FTD (10.0 Ci/mmol) were purchased from Moravek Biochemicals (Brea, CA, USA). dNTPs were obtained from Takara Bio (Otsu, Japan). F_3_dTTP was synthesized in house. Single-stranded DNA oligonucleotides were synthesized by Nihon Gene Research Laboratories (Sendai, Japan).

### Cell culture

The human HCT116 colorectal cancer cell line derived from an adult male was obtained from the American Type Culture Collection (ATCC; Manassas, VA, USA) and cultured at 37°C with 5% CO_2_ in McCoy’s 5A modified medium supplemented with 10% fetal bovine serum (FBS). Short tandem repeat profiling was performed to confirm the origin and authenticity of the HCT116 cell line.

### Measurement of dThd, FTD and FdUrd incorporation into DNA

HCT116 cells (1×10^7^) were seeded overnight in 175-cm^2^ culture flasks. Subsequently, cells were incubated with drug mixtures containing tritium-labeled nucleosides at a final concentration of 1 μmol/l. The cells were harvested at 1, 2, 4, 10 and 24 h after treatment with each drug. DNA was extracted from cell pellets using a DNeasy Blood & Tissue kit (Qiagen, Hilden, Germany). The resulting DNA solutions were dissolved in 10 ml of Ultima Gold AB liquid scintillation fluid (Perkin-Elmer, Tokyo, Japan). Radioactivity in the samples was measured with a Tri-Carb 2900TR liquid scintillation analyzer (Perkin-Elmer), and the incorporation of tritium-labeled nucleosides into DNA was quantitated. DNA concentrations were determined using the Qubit^®^ dsDNA BR assay kit (Life Technologies, Carlsbad, CA, USA).

### Transport experiments

Nucleoside transport was evaluated by the silicone layer method (26). Briefly, cells were harvested the day before experiments began and suspended in transport medium containing 125 mmol/l NaCl, 4.8 mmol/l KCl, 5.6 mmol/l D-glucose, 1.2 mmol/l CaCl_2_, 1.2 mmol/l KH_2_PO_4_, 12 mmol/l MgSO_4_, and 25 mmol/l HEPES (pH 7.4). Cell suspensions were pre-incubated for 20 min at 37°C in transport medium, centrifuged, and the resultant cell pellets were resuspended in transport medium (pH 7.4) containing a radiolabeled nucleoside or nucleoside analog to initiate uptake. At appropriate times, 160-μl aliquots of cell suspension were withdrawn, and cells were separated by centrifugation through a layered mixture of silicone oil (SH550; Dow Corning Toray, Tokyo, Japan) and liquid paraffin (Wako Pure Chemicals Industries) with a density of 1.03 g/ml. Cell pellets were solubilized in 3 mol/l KOH and then neutralized with HCl. Next, cell-associated radioactivities were measured using a Tri-Carb 2900TR liquid scintillation analyzer. The cellular protein content was determined using the Qubit^®^ Protein assay kit (Life Technologies).

### Active recombinant protein expression and purification of TK1 and DUT

The pENTR221/TK1 and pENTR221/DUT constructs were obtained from Life Technologies. A Gateway LR reaction was performed to subclone the fusion partners into the Gateway bacterial expression vector pEXP1/DEST. Transformed BL21 Star^TM^ (DE3) pLysS cells were grown for 10 h at 37°C in LB medium containing 100 μg/ml ampicillin. Gene expression was induced by adding isopropyl β-D-1-thiogalactopyranoside (0.1 μmol/l) that was cooled to 10°C. After 18 h of induction at 25°C, bacteria were collected by centrifugation, and pellets were stored at −135°C until purification. Bacterial pellets were resuspended in 10 ml BugBuster^®^ HT Protein Extraction reagent (Merck KGaA, Darmstadt, Germany) and incubated for 30 min at room temperature. Subsequently, the extract was clarified by centrifugal filtration with a 0.4-μm filter and purified with an AKTAprime plus with a HiTrap metal chelate column (GE Healthcare, Buckinghamshire, UK).

### Analysis of TK1 substrate specificity for dThd, FTD, dUrd and FdUrd

Ten micromolar tritium-labeled nucleosides and nucleoside analogs were incubated for 5 min at 37°C in 100-μl reactions containing 150 mmol/l Tris-HCl (pH 7.6), 6 mmol/l ATP, 15 mmol/l MgCl_2_, 1% bovine serum albumin (BSA) and 20 ng recombinant human TK1. Reactions were stopped by the addition of 200 μl of methanol and clarified by ultrafiltration. After clarification, solutions were desiccated by exposure to N_2_ gas, dissolved in 100 μl H_2_O, and analyzed by radio-high-performance liquid chromatography (HPLC). HPLC was performed using a 150TR Flow System analyzer (Perkin-Elmer) and a Prominence Series HPLC system (Shimadzu Corp., Kyoto, Japan) equipped with an ODS reverse-phase column (TSKgel ODS-100V, 250 mm × 4.6 mm, 3 μm; Tosoh Corp., Tokyo, Japan), with the eluent [10 mmol/l phosphate buffer (pH 3.0):Acetonitrile = 9:1] set to a flow rate 1.0 ml/min for 15 min. This procedure enabled quantitation of residual nucleosides and the production of nucleoside monophosphates formed in the reactions just described.

### Analysis of DUT substrate specificity for dThd, FTD, dUrd and FdUrd-triphosphate

dUTPase assays were performed as previously described (27). Deoxynucleoside triphosphates (dUTP, dTTP, F_3_dTTP and FdUTP; 30 mmol/l) were incubated for 30 min at 37°C in 100-μl reactions composed of 50 mmol/l Tris-HCl (pH 7.4), 4 mmol/l MgCl_2_, 2 mmol/l 2-mercaptoethanol, 0.1% BSA and 100 ng human recombinant DUT. Reactions were stopped by the addition of 11 μl 4.2 mol/l perchloric acid per reaction. Samples were clarified by centrifugation for 10 min at 13,000 rpm. Supernatants (70 μl) were neutralized with 32 μl of 1 mol/l K_2_HPO_4_, followed by a 10-min centrifugation at 13,000 rpm. Supernatants (10 μl) were resolved by HPLC as described above to quantify residual dUTP and newly formed dUMP.

### Substrate specificity of dThd, FTD, dUrd and FdUrd-triphosphate for DNA polymerase α

Recombinant DNA polymerase α (CHIMERx, WI) activity was assayed according to the manufacturer’s recommended protocol. The substrates (dNTPs) were incubated for 30 min at 37°C with 60 mmol/l Tris-HCl (pH 7.0), 5 mmol/l MgCl_2_, 0.3 mg/ml BSA, 1 mmol/l DTT, 0.1 μmol/l, 0.5 U DNA polymerase α, fluorescein isothiocyanate (FITC)-labeled primer (5′-FITCGTAAAACG ACGCC AGT-3′), and 0.1 μmol/l synthetic DNA template (5′-TCG GACTGGCCG TCG TTTTAC-3′, 5′-TCG CACTGGCCG TCG TTTTAC-3′, 5′-TCG AACTGGC CG TCG TTTTAC-3′, or 5′-TCG TACTGGCCGTCGTTTT AC-3′). The underlined sequences represent nucleotides that were modified partially to confirm the sites where F_3_dTTP and FdUTP were inserted.

After the enzyme reactions were complete, samples were resolved by electrophoresis on an ERICA-S system (DRC, Tokyo, Japan) at 300 V for 3.5 h, and FITC-fluorescence was detected using an ImageQuant LAS 4010 imager (GE Healthcare).

### Morphological analysis of HCT116 cell nuclei treated with FTD and FdUrd

For electron microscopy studies, samples were fixed with 2% paraformaldehyde and 2% glutaraldehyde, after which they were exposed to 2% osmium tetroxide. Following dehydration with a graded ethanol series, samples were transferred to resin quetol 812 (Nisshin EM, Tokyo, Japan) and polymerized at 60°C for 48 h. The blocks were sectioned at 70 nm with an Ultracut UCT ultramicrotome (Leica Microsystems, Wetzlar, Germany), and sections were placed on copper grids and stained with 2% uranyl acetate and lead stain solution (Sigma-Aldrich, Carlsbad, CA, USA). The grids were evaluated using a JEM-1200EX transmission electron microscope (JEOL, Tokyo, Japan).

## Results

### Incorporation and elimination of dThd, FTD and FdUrd from DNA in HCT116 cells

**Figure 2:**
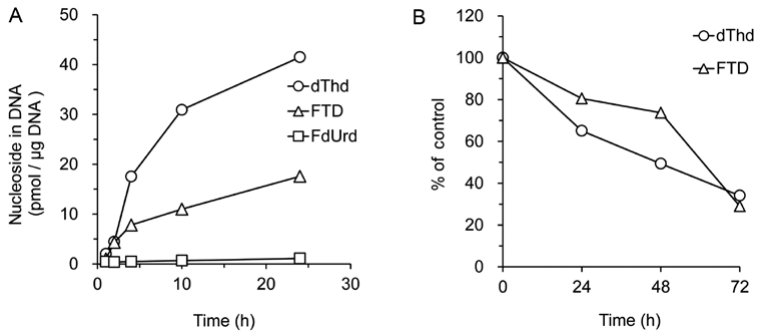
Comparison of dThd, FTD and FdUrd incorporation (A) into DNA and elimination of dThd and FTD (B) from DNA after a washout step. (A) HCT116 cells were treated for 1, 2, 4, 10 or 24 h with each compound at 1 μmol/l, and DNA incorporation was measured by LSC. (B) Following 24-h treatment with dThd or FTD, these compounds were washed out. At 24, 48 and 72 h following the washout step, the amount of dThd or FTD remaining incorporated into DNA was measured by liquid scintillation counting. Open circles, triangles and squares represent the incorporation of dThd, FTD and FdUrd, respectively.

Initially, we compared the rates of FTD and FdUrd accumulation into DNA in HCT116 cells with that of the native substrate dThd, using final nucleoside concentrations of 1 μmol/l. Fig. 2A shows the time course for nucleoside accumulation at 1, 2, 4, 10 and 24 h post-exposure. Tritium-labeled nucleosides were used as substrates and were quantified by liquid scintillation counting (LSC). dThd and FTD were incorporated into DNA at comparable levels at the 1- and 2-h time-points. dThd incorporation increased linearly until 10 h, while saturation of FTD incorporation occurred by 4 h, reaching approximately half that observed with dThd (dThd, 41.5 pmol/μg DNA; FTD, 17.6 pmol/μg DNA). However, the level of FdUrd incorporation into DNA was relatively low. For example, after a 24-h incubation, FdUrd incorporation was one sixteenth of that observed with FTD (FdUrd, 1.1 pmol/μg DNA), which is close to the limit of detection.

Next, we compared the susceptibility of FTD to elimination from DNA with that of dThd. In this assay, cells were treated with 1 μmol/l dThd and FTD for 24 h, after which drug-free medium was added and the cells were incubated for an additional 24, 48 or 72 h. Fig. 2B shows the residual ratios of dThd to FTD at 24, 48 and 72 h after the washout steps. Both dThd and FTD incorporation into DNA decreased gradually after the washout step, and only minor differences were observed between dThd and FTD (dThd 34.1%, FTD: 29.0%). We also attempted to assess FdUrd elimination from DNA after the washout step; however, it was nearly undetectable in this assay and could not be evaluated.

### Intracellular uptake of dThd, FTD, dUrd and FdUrd in HCT116 cells

**Figure 3:**
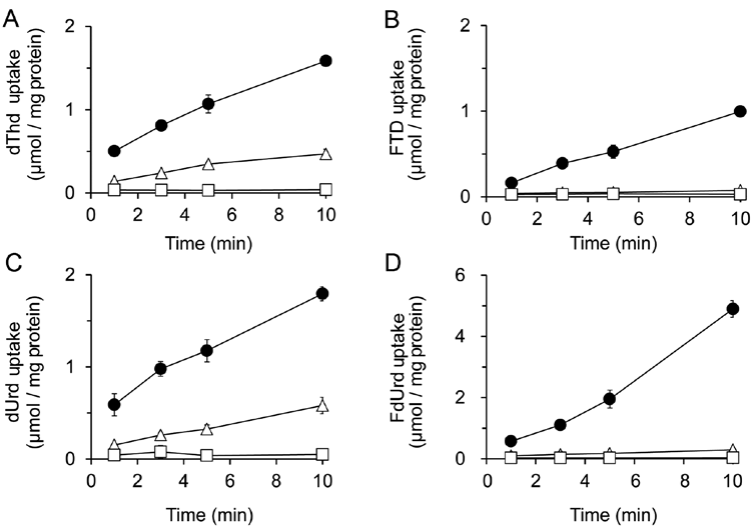
Comparison of uptake levels of [^3^H]dThd (A), [^3^H]FTD (B), [^3^H]dUrd (C) and [^3^H]FdUrd (D) and the effects of nucleoside transporter inhibitors. The uptake of 1 μmol/l (1 μCi/ml) dThd, FTD, dUrd and FdUrd was measured for 1, 3, 5 and 10 min by LSC. HCT116 cells were pre-incubated for 10 min at 37°C in transport buffer (pH 7.4) before initiating the uptake assay. Closed circles, open triangles and squares represent cellular uptake with a control, 1 μmol/l NBMPR and 10 μmol/l DPM, respectively. Each result represents the mean ± SD (n=3).

To monitor the transport of FTD and FdUrd into the cytoplasm and their intracellular uptake, the inhibitory effects of the nucleoside transporter inhibitors 6-[(4-nitrobenzyl) thio]-9-(β-D-ribofuranosyl) purine (NBMPR) and dipyridamole (DPM) were analyzed and compared to the native substrates dThd and dUrd, respectively (Fig. 3). The concentration of inhibitors used was based on the concentration of NBMPR required to inhibit ENT1, or that required for DPM inhibition of both ENT1 and ENT2 (28).

After incubating HCT116 cells with dThd or FTD for 10 min, we found that the inhibitory effect of 1 μmol/l NBMPR was less than that of 10 μmol/l DPM. For instance, the levels of cellular dThd uptake at 10-min post-treatment with a vehicle control, 1 μmol/l NBMPR, or 10 μmol/l DPM were 1.59±0.06, 0.47±0.06 or 0.04±0.01 nmol/mg protein, respectively, while those for FTD uptake were 1.00±0.04, 0.07±0.01 and 0.03±0.02 nmol/mg protein, respectively (Fig. 3A and B).

HCT116 cells were also incubated with dUrd and FdUrd for 10 min, and inhibition by 1 μmol/l NBMPR was again lower than that by 10 μmol/l DPM. The levels of dUrd uptake at 10-min post-incubation with a vehicle control, 1 μmol/l NBMPR, or 10 μmol/l DPM were 1.80±0.08, 0.58±0.09 and 0.05±0.01 nmol/mg protein, respectively, and those of FdUrd were 4.90±0.27, 0.29±0.07 and 0.03±0.00 nmol/mg protein, respectively (Fig. 3C and D). These results indicate that all nucleosides tested in these experiments could be recognized and transported into cells by both ENT1 and ENT2.

### Analysis of TK1 affinities for dThd, FTD dUrd and FdUrd

To evaluate the substrate specificity of FTD, dUrd and FdUrd for TK1, recombinant human TK1 was incubated with tritium-labeled substrates. The K_m_ values determined for each tested nucleoside revealed that dThd analogs had a higher affinity for TK1 than for dUrd analogs (Table I). The V_max_ values of dThd-based compounds exceeded those of the dUrd-based compounds. FTD and FdUrd showed higher affinities compared to the native form of these nucleosides, namely, dThd and dUrd.

To evaluate the catalytic efficiency of TK1 in phosphorylating dThd, FTD, dUrd and FdUrd, k_cat_/K_m_ ratios were calculated. The k_cat_/K_m_ values of dThd and its analog FTDs tended to be higher than those of dUrd and its analog FdUrd (Table I). TK1 showed higher catalytic efficiency in phosphorylating FTD than it did with dThd, and the catalytic efficiency of FTD phosphorylation by TK1 was ~4-fold higher than that of FdUrd.

### Substrate specificity of DUT for the triphosphate forms of dThd, FTD, dUrd and FdUrd

Pyrimidine triphosphate incorporation into DNA is strictly regulated by DUT (29,30) and high DUT expression is reported in various types of cancer, suggesting that DUT may confer resistance to 5-fluoropyrimidines (24,30). To determine whether FTD is substrate of DUT, we performed enzymatic assays with recombinant human DUT and either FdUTP or F_3_dTTP (Table II). Although DUT efficiently converted dUTP and FdUTP to their monophosphate forms (dUMP and FdUMP, respectively), dTTP and F_3_dTTP were not found to be substrates for DUT.

### Substrate specificity of DNA polymerase α for dThd, FTD, dUrd and FdUrd-triphosphate

**Figure 4:**
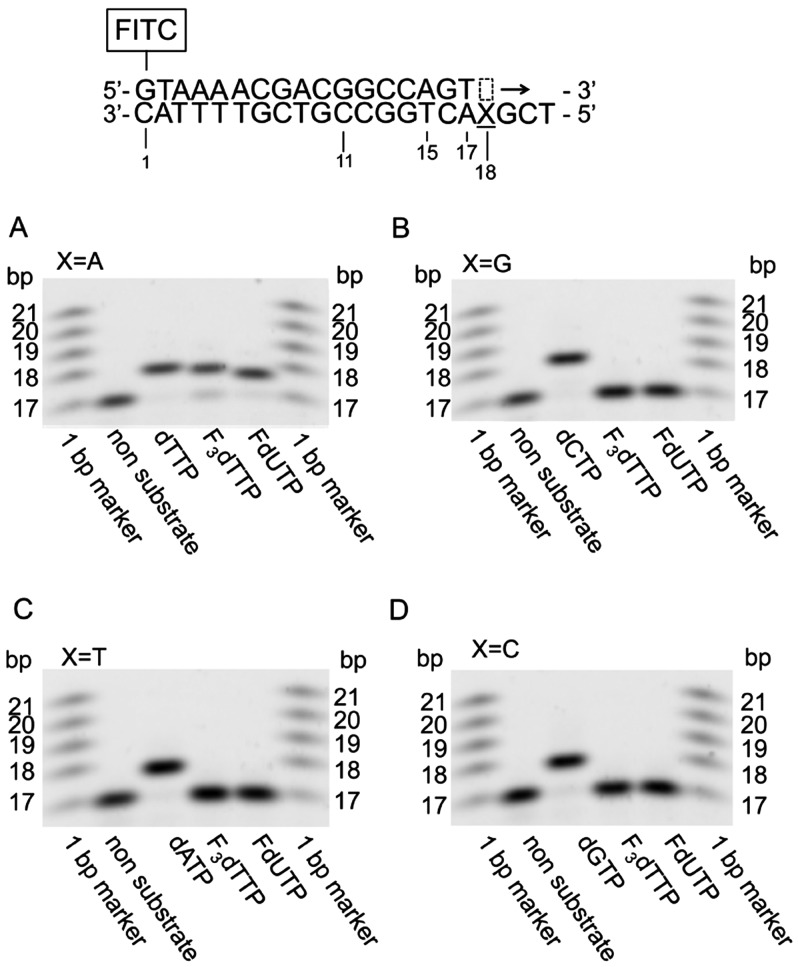
Substrate specificity of DNA polymerase α for incorporating the triphosphate forms of FTD, dUrd and FdUrd into the complement DNA strand at sites matching adenine (A), guanine (B), thymine (C), or cytosine (D). Substrates were incubated for 30 min at 37°C in reaction buffer containing a single-stranded DNA oligonucleotide hybridized to an FITC-labeled primer (0.1 μmol/l) and DNA polymerase α. After reactions were completed, the samples were denatured and electrophoresed on a denaturing acrylamide gel at 300 V for 3.5 h.

Next, we investigated whether F_3_dTTP and FdUTP serve as substrates for DNA polymerase α, the responsible enzyme for catalyzing DNA replication. We analyzed extension reactions by DNA polymerase α using dNTPs (as control substrates) and the triphosphate forms of dUrd, FTD and FdUrd, and a synthetic single-stranded DNA template (Fig. 4). F_3_dTTP and FdUTP, dThd and dUrd were incorporated into the nascent strand at sites matching adenine on the template strand, but not at guanine, cytosine or thymine sites.

### Morphological analyses of HCT116 cells treated with FTD and FdUrd

**Figure 5:**
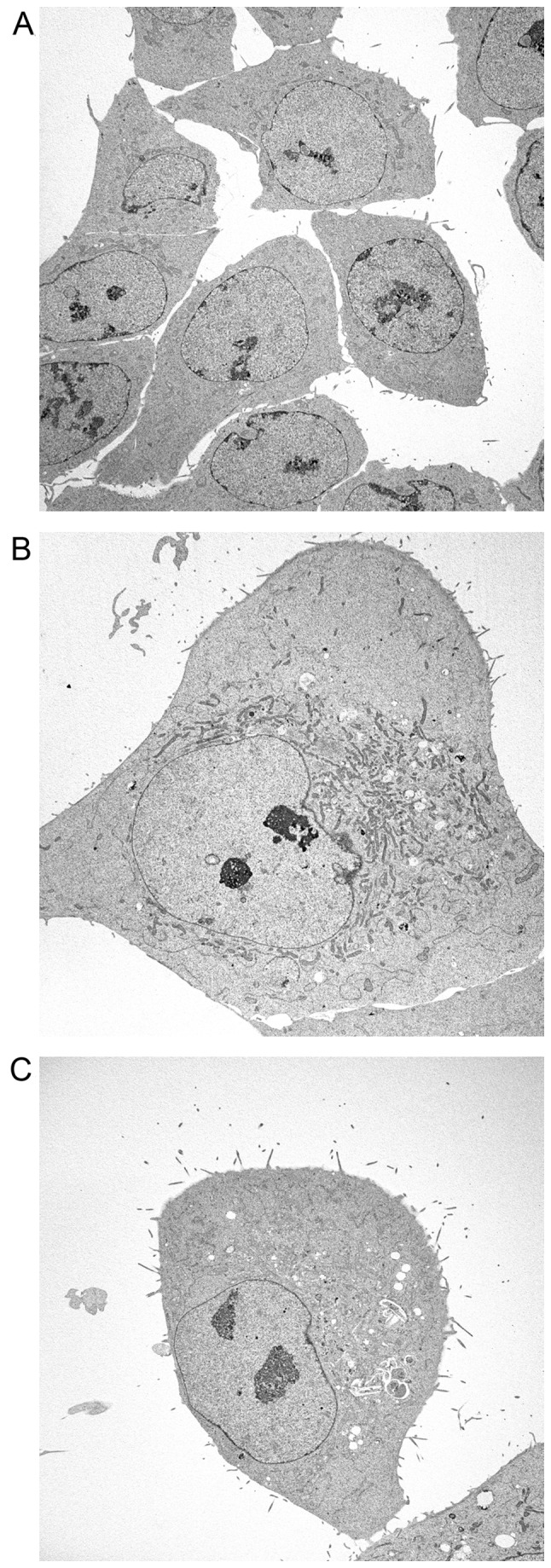
Electron microscopy analysis of untreated HCT116 cells (A), or HCT116 cells treated with FTD (B) or FdUrd (C). Cells were treated in 60-mm culture dishes with 6 μmol/l FTD or 3 μmol/l FdUrd for 72 h, using a concentration found capable of 50% growth inhibition (data not shown) and subsequently fixed. After dehydration by ethanol treatment, samples were transferred to resin and polymerized. The acceleration voltage of an electron microscope was set at 80 kV, and observations were performed under ×714 magnification.

Finally, to compare DNA incorporation data with observable phenotypes, we performed morphological analyses of HCT116 cells treated with FTD and FdUrd by electron microscopy (Fig. 5). Swollen nuclei and structural abnormalities of nucleoli were observed in both FTD- and FdUrd-treated cells (relative to untreated control cells), with the degree of alteration caused by FTD exposure much stronger than that caused by FdUrd. Furthermore, the perinuclear heterochromatin content was decreased by both FTD and FdUrd treatment.

## Discussion

**Figure 6:**
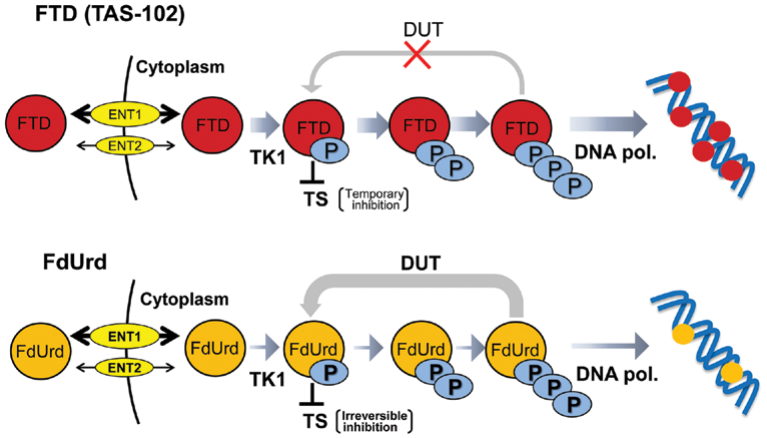
A potential mechanism of FTD and FdUrd incorporation into DNA via the dThd salvage pathway. In this model, both FTD and FdUrd are transported by both ENT1 and ENT2. The catalytic efficiency of TK1 for FTD phosphorylation is higher than that for FdUrd. FdUrd-triphosphate is degraded by DUT, while FTD-triphosphate is not a DUT substrate. Consequently, FTD incorporates into DNA effectively, compared with FdUrd.

In the present study, we demonstrated that FTD is incorporated into DNA with greater efficacy than is FdUrd (Fig. 6), which resulted from differences in the affinity of TK1 and DUT for these nucleosides. These differences may overcome resistance to FdUrd. The catalytic efficiency of FTD phosphorylation by TK1 is 4-fold higher than that of FdUrd and dUrd. These results imply that TK1 may recognize FTD as dThd, while FdUrd may mimic dUrd. A similar phenomenon was observed for the substrate specificity of DUT. The triphosphate forms of both dUrd and FdUrd were degraded to their monophosphate forms by DUT, while the triphosphate forms of dThd and FTD did not serve as DUT substrates. As a result, FTD-triphosphate was not only produced at a high level, but was also retained for a relatively long duration in DNA (16), compared with FdUrd. In contrast, the monophosphate form of FdUrd may accumulate at a high level, and TS inhibition may be potentiated by DUT.

Regarding the relationship between DNA incorporation and cytotoxicity, we previously reported that one factor promoting higher FTD incorporation than FdUrd incorporation is TS inhibition (17). Indeed, FdUMP derived from FdUrd irreversibly inhibits TS through the formation of a covalent bond, whereas F_3_dTMP inhibits TS in a reversible manner. Short-term TS inhibition by F_3_dTMP is not sufficient to cause cytotoxicity, but may still deplete dTMP and dTTP, enabling F_3_TTP to be incorporated into DNA at a higher level.

FTD is resistant to degradation by DUT, suggesting that TAS-102 could be effective against tumors resistant to 5-FU because of elevated DUT expression (31). In addition, several investigators have shown that patients with high TK1 expression have a relatively poor prognosis with some tumor types (32). These findings indicate that TK1 overexpression represents a cancer-specific pathway and that TAS-102 treatment can improve the poor prognosis of metastatic colorectal cancer patients who are refractory to standard chemotherapies, including 5-FU.

From the viewpoint of FTD resistance, the downregulation of nucleoside transporters and TK1 involved in the dThd salvage pathway results in ineffective therapy against cancer cells (23). These factors indicate that FTD-resistant cells may rely on *de novo* dThd synthesis as an alternative means of dThd production. A TS inhibitor cocktail involving 5-fluoropyrimidines and/or anti-folates may be a good choice in treating FTD-resistant cancer. Modulating dThd synthesis by 5-FU and FTD could be an effective cancer therapy.

The DNA repair system removes DNA damage sites from the genome, making it one of the determinants of 5-fluoropyrimidine efficacy (33). We could not analyze FdUrd efflux from DNA because of its low level of DNA incorporation. However, the elimination ratio of FTD was similar to that observed with the natural substrate dThd. This phenomenon implies that FTD is stably incorporated into DNA at the same level as dThd. It is reported that uracil DNA glycosylases, thymine DNA glycosylase, and the methyl-CpG binding domain 4 protein recognize 5-FU (but not FTD) and that they may contribute to FTD incorporation into DNA. However, further studies should be performed to confirm the associated FTD antitumor mechanisms at play following DNA incorporation, including potential involvements of DNA repair systems.

In conclusion, evidence presented here suggests that TAS-102 consisting of FTD and TPI is the first dThd-based antitumor agent whose main mechanism is the inhibition of DNA incorporation itself. Further studies regarding mechanisms occurring after DNA incorporation, as well as combination therapies with other antitumor agents may be needed to improve targeted cancer chemotherapy.

## Figures and Tables

**Table I:** The affinity of TK1 for dThd, FTD, dUrd and FdUrd.

Substrate	K_m_ (μmol/l)	V_max_ (pmol/min/ng protein)	k_cat_ (min)	k_cat_/K_m_ (x10^6^/min·mol)
dThd	1.16	0.70	17.78	15.28
FTD	2.34	0.93	10.26	19.26
dUrd	4.23	0.30	7.65	1.81
FdUrd	3.33	0.51	12.97	3.86

Tritium-labeled nucleosides were incubated for 5 min at 37°C in a 100-μl reaction buffer containing human TK1. Input substrate nucleosides and nucleotides produced by TK1 were analyzed by HPLC.

**Table II:** Substrate specificities of triphosphate form of dThd, FTD, dUrd and FdUrd for DUT.

Substrate	Production of 5′-monophosphate (pmol/min/100 ng protein)
dTTP	N.D.
F_3_dTTP	N.D.
dUTP	41.4
FdUTP	34.8

Thirty micromolar triphosphates were incubated for 30 min at 37°C in buffer containing 100 ng recombinant dUTPase. Samples were analyzed by HPLC to quantify triphosphate and monophosphate formation. N.D., not detectable.

## Abbreviations

5-FU: 5-fluorouracil
DPM: dipyridamole
dThd: deoxythymidine
dUrd: deoxyuridine
dUMP: deoxyuridine-monophosphate
dUTP: deoxyuridine-triphosphate
DUT: deoxy UTPase
FTD: trifluridine
F_3_dTMP: FTD-monophosphate
F_3_dTTP: FTD-triphosphate
FITC: fluorescein isothiocyanate
FdUrd: 2′-deoxy-5-fluorouridine
FdUMP: FdUrd-monophosphate
FdUTP: FdUrd-triphosphate
hENT: human equilibrative nucleoside transporter
HPLC: high performance liquid chromatography
LSC: liquid scintillation counting
NBMPR: 6-[(4-nitrobenzyl) thio]-9-(β-D-ribofuranosyl) purine
TK: dThd kinase
TS: thymidylate synthase
